# X-ray computed tomography scanning schemes and reconstruction capabilities at the BAMline (BESSY II)

**DOI:** 10.1107/S1600577526001177

**Published:** 2026-02-20

**Authors:** H. Markötter, M. Sintschuk, G. Bruno

**Affiliations:** a Bundesanstalt für Materialforschung und -Prüfung (BAM) Unter den Eichen 87 12205Berlin Germany; b Physikalisch-Technische Bundesanstalt (PTB) Abbestraße 2-12 10587Berlin Germany; Australian Synchrotron, Australia

**Keywords:** synchrotron X-ray imaging, computed tomography, preview reconstruction, CT scan scheme, software workflow

## Abstract

The BAMline at the BESSY II synchrotron (Berlin, Germany) is a multi-purpose beamline that is used for computed tomography, among other techniques. For more than 20 years it has been used in various scientific fields to access and analyze internal structural features such as porosity, cracks or delamination. The general trend of tomographic X-ray imaging is moving towards *in situ* and *operando* studies to access the above-mentioned features under the most realistic conditions. This article presents the X-ray computed tomography (XCT) capabilities and the software suite, such as live reconstruction available to imaging users. Direct XCT reconstruction plays an important role in the quality control of the performed experiments and is particularly crucial for *in situ* and *operando* experiments. The BAMline now offers a sequence-based step-scan XCT that enables preview reconstruction *during* the scan to gain early insight into the scanned sample even while the scan is in progress. In addition, the sequence-based scheme includes lateral shifts, thereby mitigating ring artifacts and improving image quality. Finally, since XCT scan parameters, such as sample–detector distance and beam energy, are of utmost importance, we devised calculation software allowing control of monochromators to set the desired beam spectrum.

## Abstract 2

An X-ray computed tomography scanning approach available at the BAMline effectively removes ring artifacts and enables real-time preview reconstruction through sequence-based step-scanning. Additionally, integrated X-ray spectral calculation software streamlines beamline adjustments for optimized imaging performance.

## 1. Introduction

X-ray computed tomography (XCT) is an important standard technique in the field of non-destructive testing and medical imaging, since it provides detailed internal structures of objects (Banhart, 2008 ▸). Among the various XCT techniques, synchrotron X-ray computed tomography (SXCT) stands out due to its ability to exploit a high X-ray flux and achieve high resolution down to ∼1 µm. A superior image quality is made possible using monochromators, which enable beam-hardening-artifact-free reconstructions. Due to beam coherence, the parameter sample–detector distance as well as beam energy are the most important when image contrast needs to be optimized for further image analysis (Paganin *et al.*, 2002 ▸). However, despite these advantages, there remains a trade-off between signal-to-noise ratio (SNR) and time resolution, which must be carefully managed to optimize imaging results towards dynamic processes.

Hence, for each individual sample analyzed using SXCT, it is crucial to determine the appropriate imaging parameters; for example, beam energy, sample–detector distance and pixel size, to name a few. These parameters significantly influence the characteristics and quality of the resulting images. Varying them is therefore key to optimizing the results. This usually leads to several test scans that are later of no use. Besides, non-perfect normalization during image processing can lead to ring artifacts. These can be mitigated through post-processing techniques (Vo *et al.*, 2018 ▸; Gürsoy *et al.*, 2014 ▸), which do not fully work in every case.

Off-standard scanning schemes have evolved to address these and various other challenges in XCT (Kaestner *et al.*, 2011 ▸; Köhler, 2004 ▸). Some of these scanning schemes only pay off if the time for stage rotation is shorter than the exposure times of the individual projections. However, this is usually not the case, and adapted variable scanning schemes are a better choice.

Therefore, a sequence-based scanning technique has been set up at BAMline (at the BESSY II synchrotron radiation facility). This allows effective ring artifact handling, and provides a preview reconstruction, which allows an early quality evaluation and intervention to optimize the imaging parameters. This procedure is not only interesting for synchrotron beamlines but also for laboratory-based XCT systems. In this article, we present the scanning schemes available to users at the BAMline. We detail the set of software tools offered at the XCT endstation^1^, which are designed to enhance the user experience and improve imaging outcomes. These tools, also available via GitHub^2^, provide comprehensive solutions for various imaging challenges, ensuring high-quality reconstructions and efficient data processing.

## 2. BAMline

**Figure 1:**
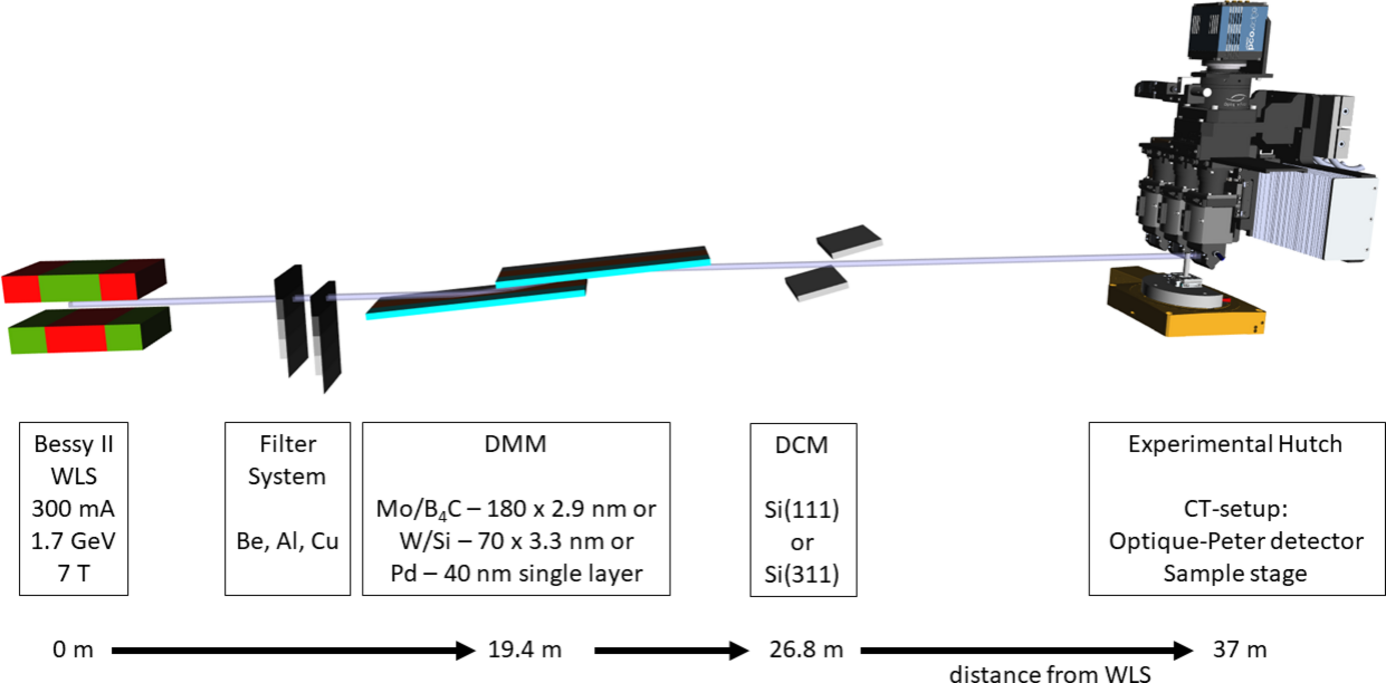
Beamline layout of the BAMline. A 7 T wavelength shifter (WLS) generates a broad X-ray spectrum, which is tailored to the specific needs via a filter set, a double-multilayer monochromator (DMM) and a double-crystal monochromator (DCM). (Component dimensions and distances are not to scale.)

BAMline is a multi-purpose beamline located at BESSY II, HZB, Berlin, Germany, a third-generation synchrotron, which operates at an electron energy of 1.7 GeV and a ring current of 300 mA (see Fig. 1 ▸). In 1999, BAMline was installed and later equipped with an imaging endstation (Görner *et al.*, 2001 ▸; Rack *et al.*, 2008 ▸; Markötter *et al.*, 2022 ▸; Markötter *et al.*, 2023 ▸). The experimental hutch with several different endstations starts ∼37 m from the wavelength shifter (WLS). The 7 T WLS produces an energy spectrum from ∼8 to 60 keV. This broad spectrum is modified with optical elements to obtain a monochromatic beam. These optical elements comprise a filter system containing two filter sets with Be, Al and Cu foils of various thicknesses, a double-multilayer monochromator (DMM) with three different areas (Mo/B_4_C, W/Si and Pd), each with different characteristics, and a double-crystal monochromator (DCM) with Si(111) or Si(311) crystals.

The DMM and DCM can be used individually or in combination. For XCT experiments, usually only the DMM W/Si is used, since it offers an optimal ratio between monochromaticity and X-ray flux. Setting up all the optical elements can be tricky and cumbersome without experience and knowledge of the components. It is similar to the choice of the optimal CT-scan parameters. Therefore, various software have been set up to facilitate beam tailoring and CT-scan parameters.

**Figure 2:**
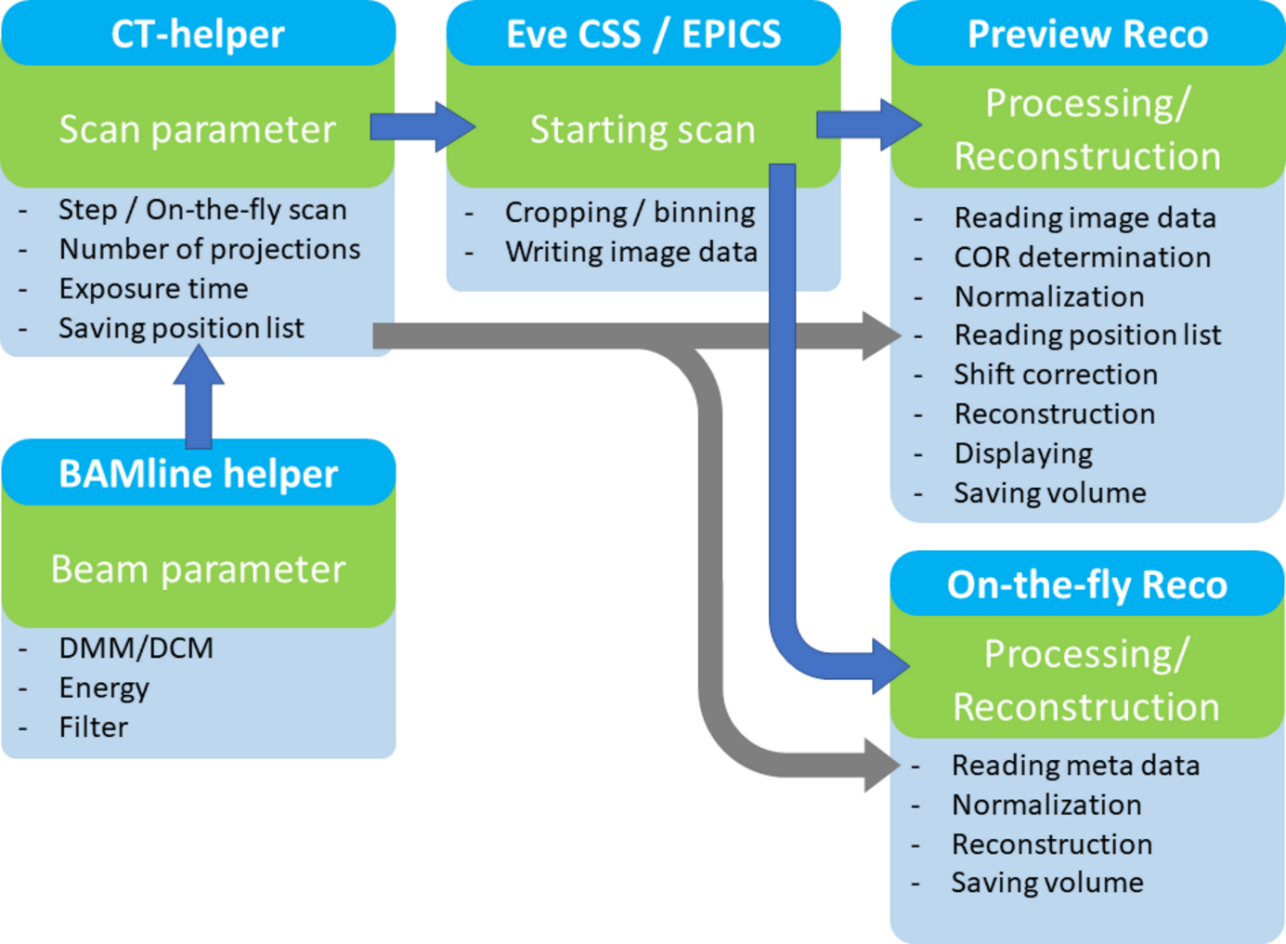
Software workflow scheme. The software constellation that facilitates the implementation of SXCT at the BAMline: *BAMline helper*, *CT helper*, *eveCSS/EPICS* and *Preview Reco* or *On-the-fly Reco*. Workflow indicated by blue arrows; metadata flow indicated by gray arrows.

Fig. 2 ▸ provides an overview of the software workflow that uses several Python solutions from preparation of XCT experiments to data reconstruction. These consist of setting up the beam parameters, defining the XCT scan parameters, starting the scan, and image processing and volume reconstruction.

*BAMline helper* is used to set up the beam. It uses the Python *XRT* package (Klementiev & Chernikov, 2014 ▸) to calculate the X-ray beam spectrum after passing the optical elements with their respective parameters, *i.e.* choice of monochromator (combination), energy, filter combination, beam offset and many more. *CT helper* allows the user to define which type of CT-mode and with which parameters, *e.g.* number of projections or exposure time, the sample is scanned. The actual beamline control is performed with *CS-Studio* (Phoebus), which offers a graphical user interface for *EPICS*. The scans are written in script style using *eveCSS*, which was developed based on *Control System Studio* (*CSS*) at Physikalisch Technische Bundesanstalt (PTB), Berlin-Adlershof, Germany. If the sample is scanned in a step-scan manner, *Preview Reco* offers the user a live reconstruction of the XCT data collected so far. In the case of an on-the-fly XCT scan, the data are reconstructed after the scan with the *On-the-fly Reco* program. Both reconstruction programs are realized with in-house Python code based on the reconstruction packages *tomopy* and *astra-toolbox* (van Aarle *et al.*, 2016 ▸; Gürsoy *et al.*, 2014 ▸). In the following sections, each program is described in detail.

## 3. BAMline helper

When working with the various optical elements at the beamline, for example the DMM option with W/Si or Mo/B_4_C multilayers in Bragg reflection or with the Pd single layer in total reflection, estimation of the beam spectrum is not easy. If the sample contains a certain element with absorption edges close to the beam energy used, knowledge of the beam spectrum is crucial, since an overlap between the absorption edge and the beam energy (*e.g.* with d*E*/*E* ≃ 3–4% for the DMM) would cause reconstruction artifacts. Therefore, *BAMline helper* has been set up—a tool to calculate the spectrum in various beamline configurations. This supports the user by setting up of all parameters for the desired beam and performing experiments.

**Figure 3:**
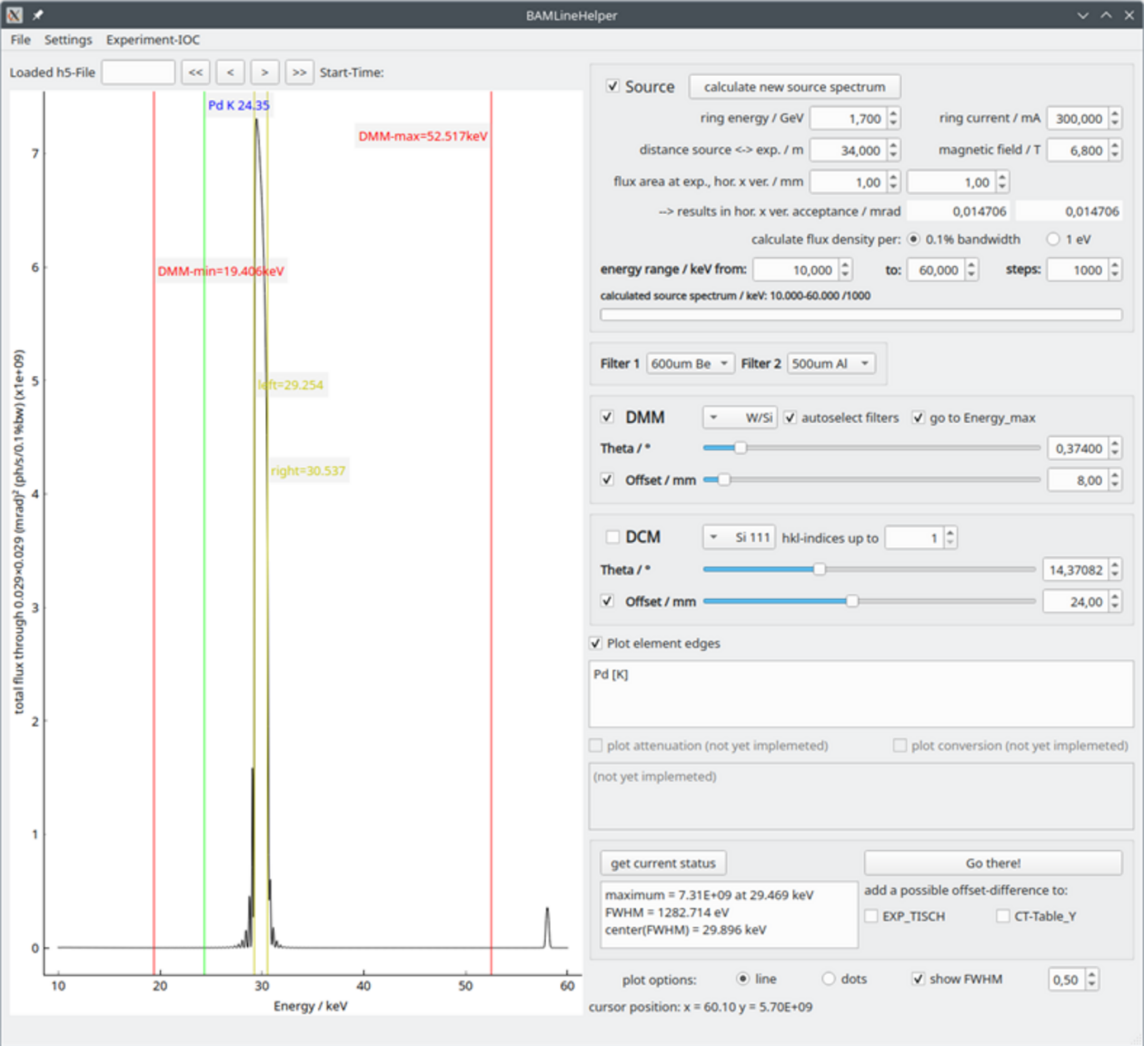
Graphical user interface of *BAMline helper*; changing the monochromator and filter settings has a direct effect on the displayed spectrum.

In Fig. 3 ▸ the main graphical user interface of *BAMline helper* is shown. From top right to bottom right: parameters are arranged for setting up the WLS spectrum, the filter settings, the DMM as well as DCM settings and plotting of absorption edges. At the very bottom, stage positions can be read from their current positions into *BAMline helper* or the position settings in *BAMline helper* can be sent to the stages. On the left-hand side, the calculated spectrum is plotted. A change of each parameter has an instant effect on the graph; only calculation of the original source spectrum (WLS) takes a few seconds if its parameters have been changed.

As a first step, the source spectrum must be calculated. To focus on the desired energy range with the required precision, the energy range and number of steps (data points) can be defined. The detailed settings of distance, ring energy, ring current and magnetic field are prefilled with the values of BESSY II/BAMline but can be adjusted if desired (*e.g.* adapted to beamlines at a bending magnet).

The WLS spectrum serves as a basis that is subsequently tailored by the settings for the filter set, as well as the DMM and/or DCM. For the DMM, there is the option of the W/Si multilayer, the Mo/B_4_C multilayer and the Pd single layer. Each can be set to a specific energy that corresponds to a specific angle of incidence (theta).

The longitudinal travel range of the second mirror in the beam direction is limited. With a fixed height position of the second DMM mirror, the so-called beam offset, only the angles of incidence (theta) within a certain range lead to a correct beam propagation (see Fig. 1 ▸, DMM). This angular range is equivalent to an energy range. Therefore, a variable height position of the second mirror is necessary to access a wide angular range. As a consequence, a certain beam offset allows a certain energy range (*e.g.* 16 mm offset → 9.5–23 keV; 8 mm offset → 19.4–52.5 keV, *etc*). This energy range is depicted in the plot by the red lines in Fig. 3 ▸, left, ‘DMM-min’ and ‘DMM-max’.

The DMM coating characteristics are prefilled but editable. At higher energies, the angle of incidence (here theta) is smaller and, above a certain point, low energy photons are totally reflected at the DMM surface. To avoid this phenomenon and maintain monochromaticity, the choice of filters is adapted to the beam energy. Filters are automatically chosen by *BAMline helper* if the corresponding check box is selected, *i.e.* ‘autoselect filters’.

The functional relation between beam energy and sample transmission does not apply in the presence of absorption edges of the sample elements, as the transmission can be drastically lowered to within a narrow energy range (a few tens of eV). However, to facilitate the choice of the energy, the absorption edges of elements can be superimposed on the plot (X-ray flux versus *E*); for example, the palladium *K*-edge is plotted with a green vertical line in Fig. 3 ▸ (‘Pd K 24.35’). For further assistance, the full width at half-maximum (‘show FWHM’) can be displayed with the center as well as the maximum (the DMM peak is not symmetrical).

When the beamline settings are well adjusted, the parameter set can be sent to the stage controls. Since some energy changes also involve a different beam offset, the XCT detector can be moved up or down simultaneously with the beam offset ‘add offset-difference to CT-Table_Y’.

## 4. CT helper

**Figure 4:**
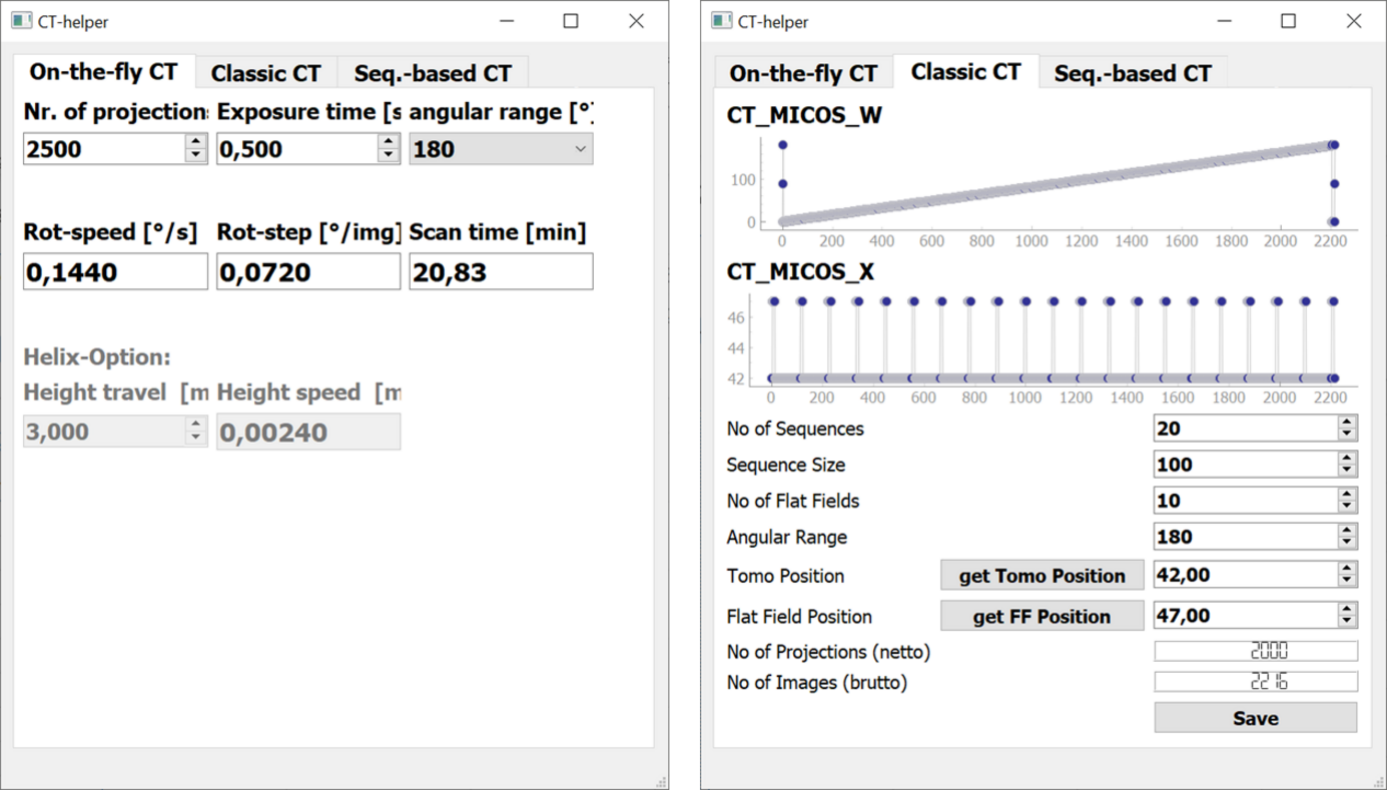
GUI of *CT helper*. The Python-based program offers tabs for the different CT modes. Left: on-the-fly CT. Right: classic step scan CT. CT_MICOS_W refers to the rotation stage and CT_MICOS_X to the lateral positioning stage.

*CT helper* is used to prepare and define CT scans. These are either ‘on-the-fly scans’ or ‘step scans’. The relatively simple ‘on-the-fly scans’ let the camera continuously record images while the rotation stage is slowly rotating at a certain speed. Flat fields are only collected before and after the scan in this mode. Therefore, it only requires the definition of the number of projections as well as the exposure time per projection and angular scan range (see Fig. 4 ▸, left). As a result, it will compute the rotation speed in degrees per second [° s^−1^], rotation step per image [° img^−1^] and net scan time [min] (without flat fields and corresponding motor travel time). These simple calculations are found on the leftmost tab.

On the second tab, called ‘Classic CT’ (see Fig. 4 ▸, right), a simple step scan can be defined, in which a list of positions for the rotation and lateral translation is created. It is defined by the angular range, the projection and flat field position, the number of projections and how often the scan will be interrupted for flat fields. The number of usable projections and the total number of images are calculated and the rotation angles as well as lateral positions are displayed. The created position list is saved as a CSV file, which is later loaded into a scan in *eveCSS*.

## 5. Sequence-based step-scan CT

**Figure 5:**
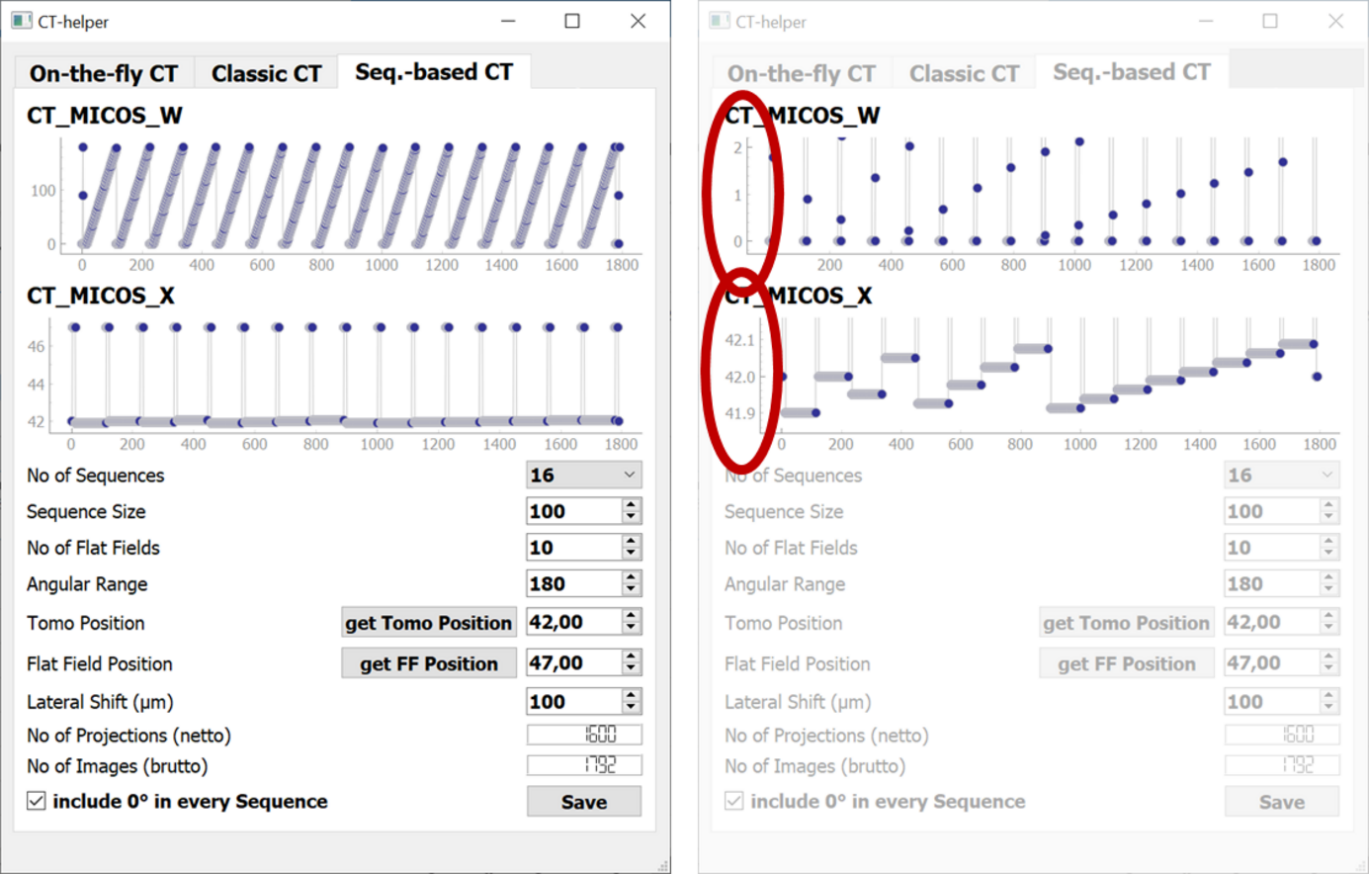
GUI for defining sequence-based step scans. Left: standard view for defining scan parameters like sequence size, *etc*. The two main stage positions, rotation stage (CT_MICOS_W) and lateral stage movement (CT_MICOS_X) are plotted. Right: the same GUI with the plot axes for rotation and lateral shift zoomed in to show the difference of the first angles and lateral shift positions for each sequence.

The preparation of the sequence-based step scans needs more input. At BAMline usually this off-standard scanning scheme is applied for step scans. The sequence-based scans consist of multiple scans from 0 to 180° (alternatively from 0 to 360°), where each scan starts with an angular offset to collect projections from angles that have not yet been recorded. Fig. 5 ▸ shows the GUI of *CT helper* in the tab for sequence-based CT. The example shown consists of 16 sequences, each with 100 projections, resulting in 1600 projections in total. Each sequence is preceded by flat fields and followed by flat fields (hence 17 × 10 flat fields), to record a more recent flat field for accurate normalization to adapt to beam instabilities caused by the DMM. Additionally, each sequence is recorded with a shifted center of rotation. In post-processing before reconstruction, the projections must be shift-corrected to match in the end. The red ellipses on the right in Fig. 5 ▸ show the starting angles (‘CT_Micos_W’) and positions (‘CT_Micos_X’) for each sequence.

Scan times of the sequence-based step-scans are not directly calculable and depend on the motor movement time and communication time with camera and motor stages. The time can easily sum up to ∼1–2 h for one scan compared with typically ∼30 min for comparable on-the-fly scans. The choice of scan type is therefore discussed in detail in the section ‘*Concluding remarks*’[Sec sec10].

This kind of procedure has several advantages. It allows a preview reconstruction to be performed during the scan, since with the first sequence the angular range of 180° is (roughly) covered. Furthermore, the technique diminishes ring artifacts (Hubert *et al.*, 2018 ▸). All advantages and details are described in the section *Preview reconstruction*[Sec sec7].

Kaestner *et al.* (2011 ▸) and Köhler *et al.* (2004 ▸) applied a scanning scheme according to the golden ratio, in which the largest gaps in the angular space are always approached and filled. With each additional projection, the angular space is scanned more and more finely. It is ideal to have projections evenly distributed across the angular range at any time of the tomography. Therefore, tomographies can be stopped at any time with a good result. The motor rotation between projections is ∼111°, quite large compared with usual rotations of ∼0.1°. Therefore, this scanning scheme is only advantageous if the stage rotation time is small compared with the exposure time of projections, otherwise the loss of time for motor movement is dominant. Therefore, we chose a sequence-based approach over the golden ratio approach.

## 6. eveCSS

*eveCSS* is a software developed by the Physikalisch-Technische-Bundesanstalt (PTB) for the control of beamlines/laboratories and has also been used at BAMline for five years. Depending on the scan type, either the rotation speed or the position list is taken from *CT helper*. Besides exposure time and angular range, many other parameters can be defined in *eveCSS*, like the naming of folder structure, *etc*. On-the-fly scans are saved in an hdf5 file including crucial metadata by *EPICS-AreaDetector*(https://github.com/areaDetector/areaDetector). The step scans are saved as a stack of tiff files, whereas their metadata are saved in a separate CSV file.

## 7. Preview reconstruction

When considering the usual workflow for classic tomographic experiments, each sample is adjusted, the scan parameters are defined, the scan is performed, and subsequently the projection data are used for volume reconstruction. In order to scan as many samples as possible in succession and not waste valuable beam time, the next scan is started immediately after completion of a scan without directly checking the success or quality of the previous one. This carries the risk of losing quality control. This problem is addressed with the software ‘*Preview reconstruction’*.

**Figure 6:**
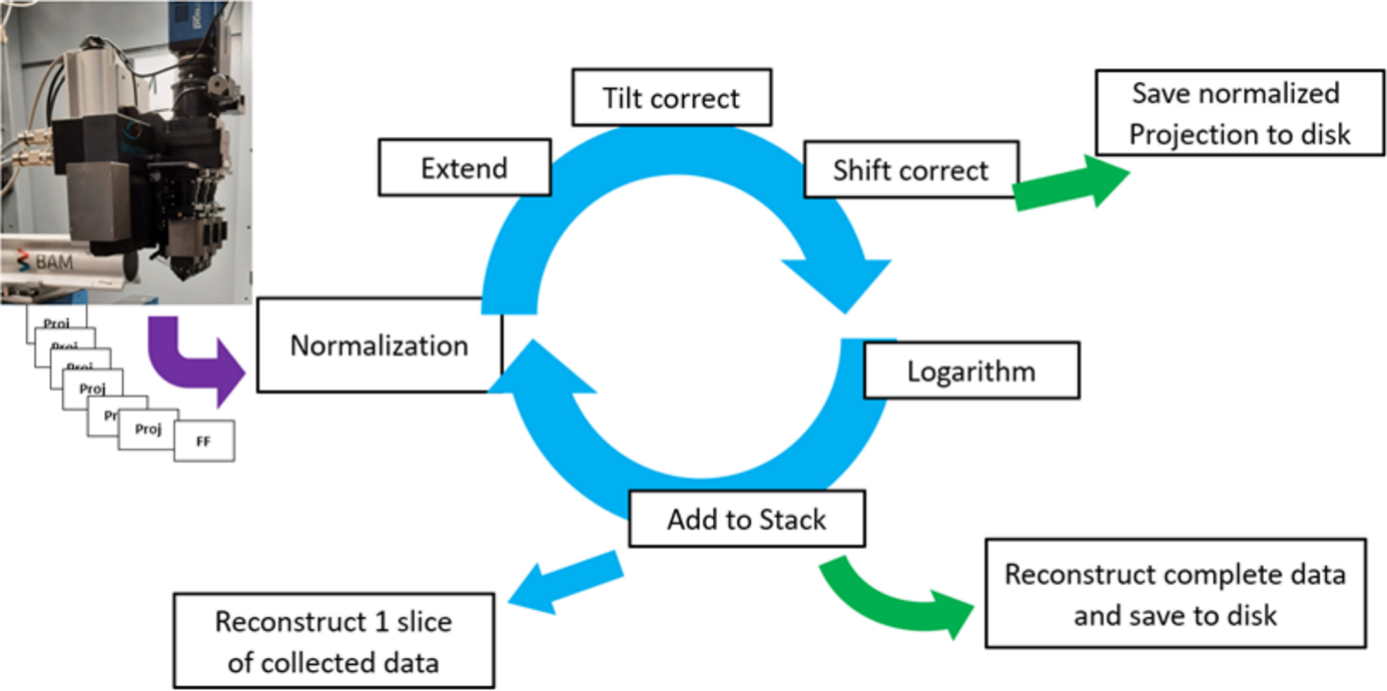
Data flow and processing scheme behind *Preview reconstruction*.

The projections of a step scan at BAMline are saved one by one in individual tiff files and are being read by the reco-program during the actual tomographic scan. The program (see the processing scheme in Fig. 6 ▸) continuously collects new image data and performs normalization and shift correction. At program start, the software defines the center of rotation and the exact pixel size by user input. Therefore, in a separate window, the projection at 0° and the mirrored projection at 180° are overlayed. The user needs to shift one to the other until a perfect match is obtained. From the shift the center of rotation is derived. In a second step, two images that were recorded at different lateral positions (*i.e.* 100 µm apart from each other) are overlayed. By perfectly matching these images, the software determines how many pixels correspond to the shift (*i.e.* 100 µm), and calculates the pixel size. Each projection is normalized with the package of flat fields recorded before the specific sequence. The package, containing ten flat fields for example, is averaged and the average is used for normalization of all projections of the subsequent sequence. In Fig. 6 ▸ the further processing scheme of the program is depicted. Each projection is normalized with the closest flat fields, the projections are subsequently extended laterally, and optionally tilt- and shift-corrected with the previously determined pixel size and position list. This is conducted with sub-pixel precision using a spline interpolation. On the one hand, those normalized and processed projections are saved to disk, but also collected in RAM after applying the negative logarithm (inverse Lambert–Beer law). For every *n*th projection, a slice is reconstructed with the data that have been collected so far and is displayed to the user.

**Figure 7:**
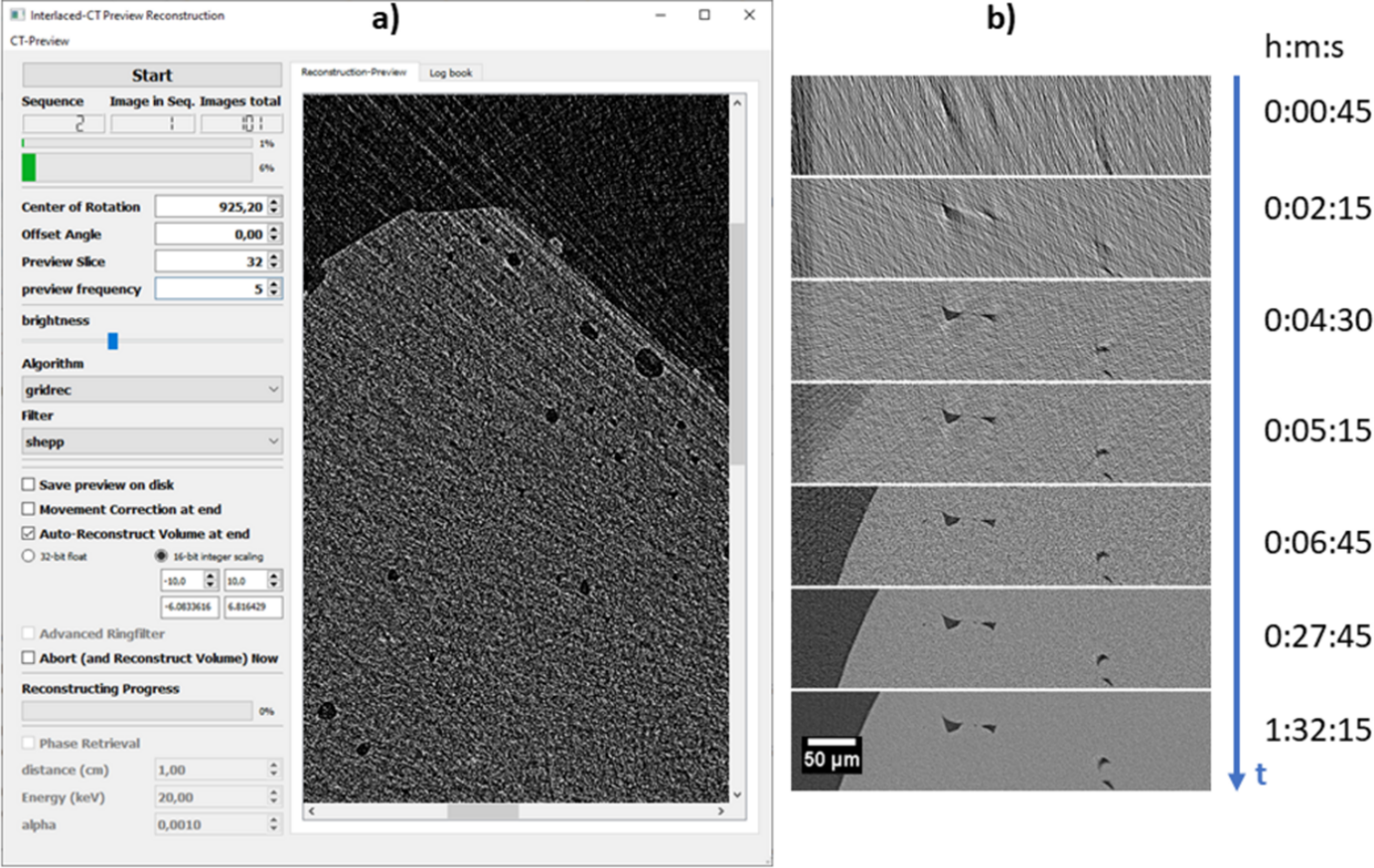
(*a*) Graphical user interface of *Preview reconstruction*. Example of a porous Al-alloy sample during the scan at 6% of the total scan duration already giving insight into the structure. (*b*) The SNR steadily improves over the scan time. Preview is shown even before collecting the first 180° rotation.

Fig. 7 ▸(*a*) shows the graphical user interface of *Preview reconstruction*. On the left-hand side, the parameters are arranged to define the reconstruction: the center of rotation (previously defined but editable), offset rotation angle, preview slice, as well as preview frequency, which defines *n*, the number of projections that are collected before the next preview reconstruction. Furthermore, the reconstruction algorithm and the filter can be chosen. The preview reconstructed slice is then displayed on the right of the GUI panel (here at 6% scan progress). In the lower left area, file-saving parameters are defined: the user can decide whether the progress of the preview should be saved and what kind of format should be used to save the volume reconstruction in the end. Either floating point precision in 32-bit or 16-bit integer can be chosen, with the respective lower and upper limits of the scaling. The progress bars of normalization of a sequence and of the whole scan are shown at the top left, whereas in the end the reconstruction progress of the whole volume is displayed by a progress bar at the bottom left. This program is intended to run in parallel to the actual scan, allowing monitoring of the scan progress and following how the results evolve with a steady increase of the SNR. Of course, the program can also be used after a scan has finished. Fig. 7 ▸(*b*) depicts the typical progress of the reconstruction results over time. In the first sequence, rough structures become more and more visible; at later stages, with increasing sequences, the reconstruction becomes more and more clear and the SNR improves.

## 8. Ring artifact handling

**Figure 8:**
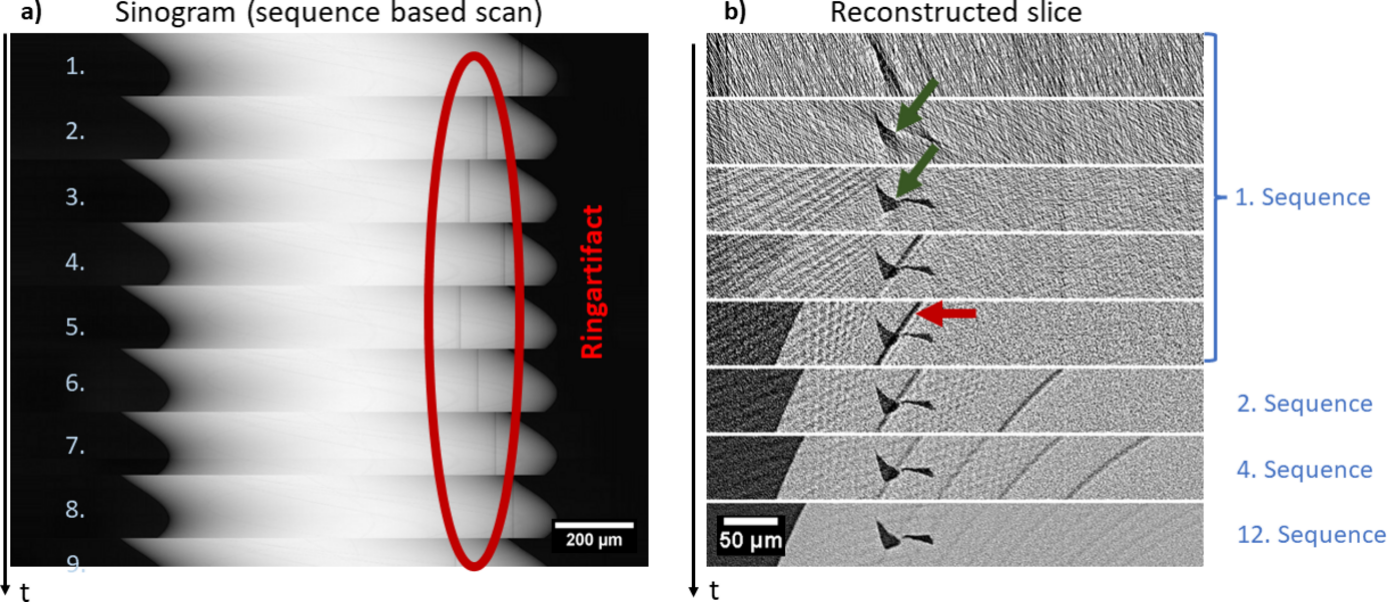
Ring artifact suppression by sequence-based scanning technique on an Al-alloy sample. (*a*) Sinogram after normalization, logarithm and shift-correction. (*b*) Reconstructed slice during the scan. The SNR increases and structures emerge (green arrows) while ring artifacts vanish (red arrow). [See the supporting information for a video.]

Ring artifacts arise by faulty detector response, *i.e.* scintillator defects or impurities with a non-linear behavior. Such artifacts lead to a subsequent improper normalization. The mathematical outcome of the filtered back projection reconstruction is a ring (half-ring if the scan range is 180°). There are numerous possibilities to filter these rings in post-processing (Vo *et al.*, 2018 ▸). Also, adapted scanning schemes can support enhancements of the image quality (Davis & Elliott, 1997 ▸). For example, we suppress ring artifacts by sample shifts, *i.e.* when dividing a tomography into sequences, the axis of rotation or the detector can be moved to a different horizontal position at each sequence. As a result, the ring artifacts are distributed [see Fig. 8 ▸(*a*), marked red] and become weaker [see Fig. 8 ▸(*b*), red arrow] with every additional sequence. This enables much more precise structural analyses of the reconstructed volumes.

**Figure 9:**
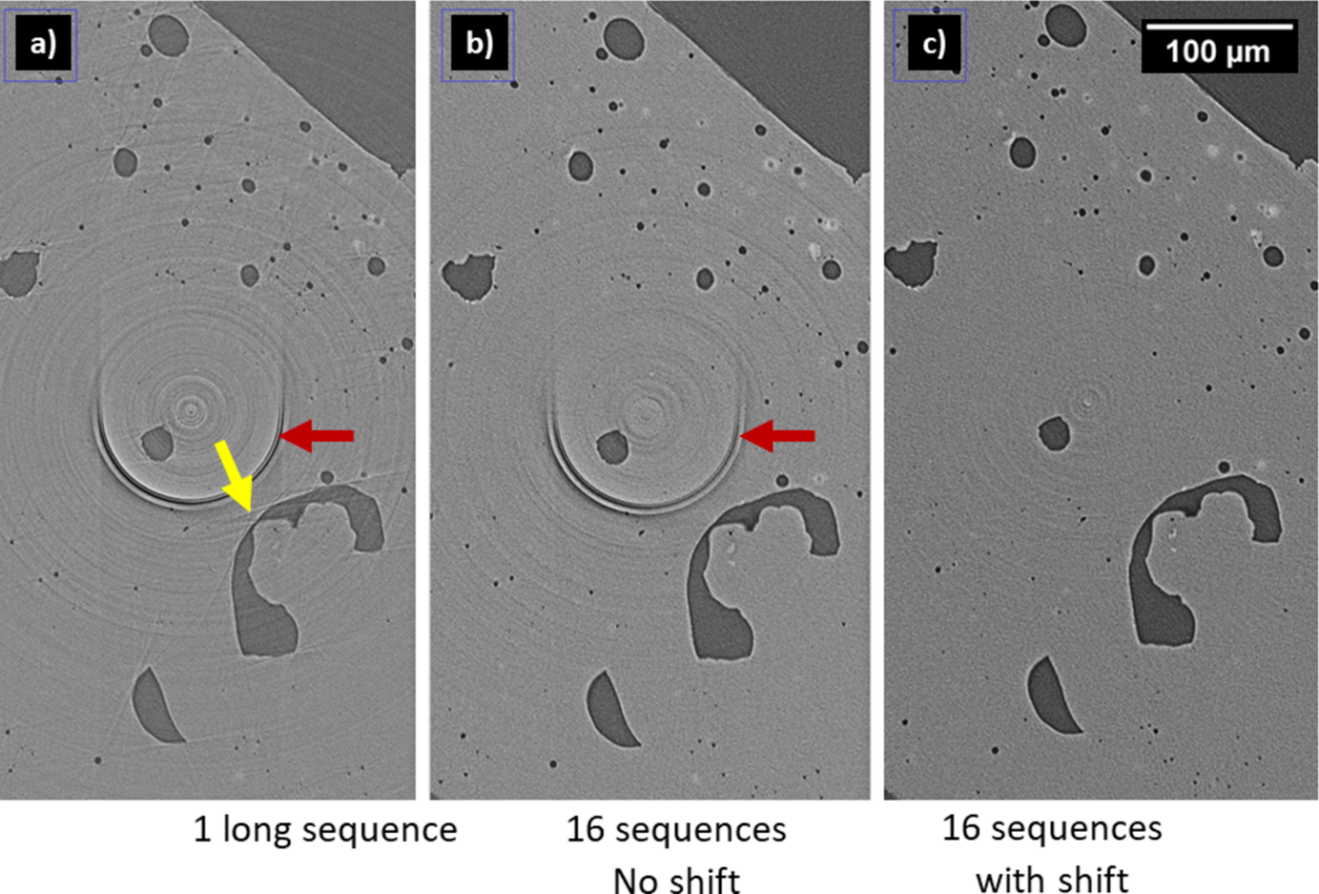
(*a*) One long sequence, (*b*) 16 sequences without shifting and (*c*) 16 sequences with shifting for ring artifact (red arrows) handling of Al-alloy sample. The sequences distribute the artifact into 16 barely visible rings. The yellow arrow marks a linear artifact caused by interruptions during flat field acquisition. [See the supporting information for a video.]

To describe the effect of such scanning schemes on a reconstructed slice, Fig. 9 ▸ displays three cases:

(*a*) The classic scanning scheme, in which 1600 projections of a step-scan were collected in one long sequence, only interrupted by flat field acquisition every 100 projections to cope with beam instabilities slowly changing the flat field profile. This reconstruction suffers from ring artifacts as well as linear artifacts. The latter arise due to the movements to the flat field positions that interrupt the tomography sequence.

(*b*) A scan comprising 16 sequences each covering a rotation of 180° with 100 projections is depicted. Each sequence starts with a slight rotation offset, so that in the end the angles are regularly sampled. As a result, the linear artifacts dis­appear.

(*c*) An additional shift of the rotation axis (sample inclusive) is introducesd at each sequence, *e.g.* of up to ±100 µm, which corresponds to ∼11% of the total field of view. This reduces ring artifacts drastically. The formerly strong ring artifact is distributed to 16 weak half rings.

The scan durations of those cases were (*a*) 59 min, (*b*) 61 min and (*c*) 61 min. From that perspective, when performing step scans there is nearly no extra time that needs to be invested into case (*c*).

## 9. On-the-fly CT scan

For high throughput an on-the-fly scanning option is available at BAMline. A sCMOS type camera (PCO edge 5.5) allows readout times of a few milliseconds and a frame rate of up to 100 full frames per second. To avoid any time delay in the detector-to-stage communication, the rotation axis is set to slowly rotate over the 180° range while the camera is continuously recording projections. With this technique, typical tomographic scans are conducted within 10–20 minutes in monochromatic mode and less than a minute in polychromatic mode. The procedure does not yet allow enhanced sequence-based scanning advantages. Potentially multiple scans with shorter exposure time, each with a lateral shift, could enable this. Currently, ring artifact handling is performed during post-processing only.

**Figure 10:**
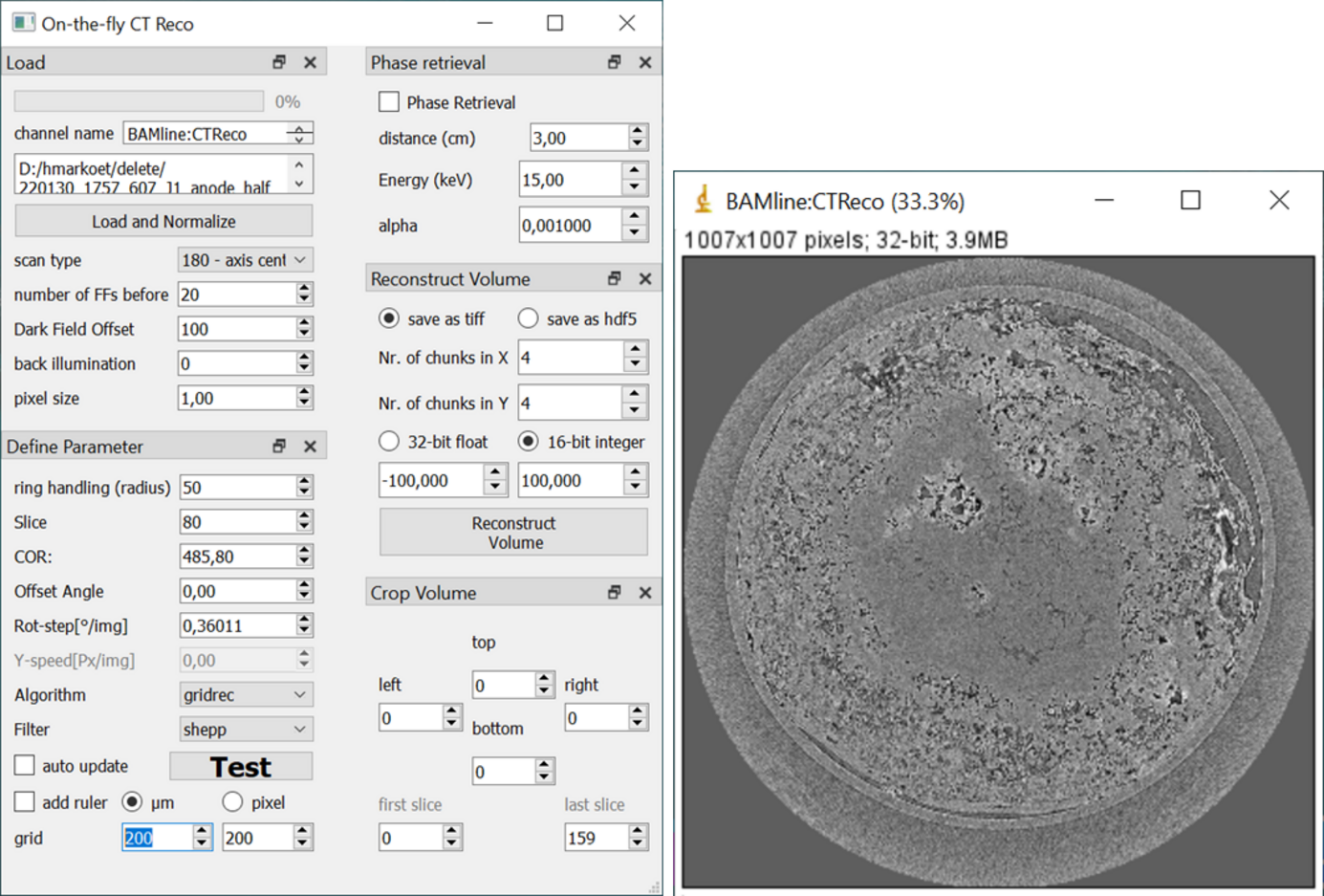
Left: graphical user interface for *On-the-fly CT reconstruction*. Right: the parameters are tested on one slice, which is sent to *ImageJ*.

Fig. 10 ▸ shows the graphical user interface of *On-the-fly CT Reco*. On-the-fly CT-data are written to an hdf5 file and read after the scan has finished with all necessary projections and flat fields. The hdf5 file contains, besides the projection data, the pixel size, rotation speed, energy, distance and other metadata that are necessary or useful. Several dock widgets give access to the various parameters, like data reading, reconstruction parameter, phase retrieval, cropping volume and volume reconstruction. The reconstructed slice is streamed via *EPICS-PVA* (Nikitin *et al.*, 2022 ▸; Rivers, 2017 ▸) to *ImageJ*. Streaming to *ImageJ* has been chosen to allow the user easy contrast settings, plotting functions and further image processing via other custom plugins.

When data are read from the hdf5 file, parameters are prefilled into the GUI, but still editable. Automatically, the center slice is normalized and reconstructed. Subsequently, parameters like center of rotation (COR) or filtering can be adjusted, and the reconstructed slice checked. For ease of handling, single slice reconstruction can activate the ‘auto update’ function that will reconstruct the slice directly when for example the value for the COR is edited, so that users can scroll via the mouse wheel to edit the COR and directly see the result in the *ImageJ* window. A further handy feature is the projection of a ruler into the reconstructed slice, showing a grid on the sample with either a certain pixel distance or a certain distance in micrometers.

If desired, phase retrieval according to Paganin *et al.* (2002 ▸) can be defined and the volume can be cropped to save disk space. The output volume can be saved as a tiff stack or an hdf5 file with defined number of chunks, both, either in 32-bit float values or as 16-bit integer values, with defined lower and upper boundaries.

## 10. Concluding remarks

In everyday CT workflow it is important to obtain feedback from an actual reconstructed slice as fast as possible in order to prevent errors or mistakes to optimize beam time and avoid unnecessary data acquisition. Furthermore, a dedicated scanning scheme for ring artifact handling substantially increases data quality.

Typical third- and fourth-generation synchrotron imaging beamlines sited at wigglers and undulators have sufficient X-ray flux that exposure times are much shorter compared with the rotation stage movement time. Therefore, the preview reconstruction is only of limited use, as a complete scan usually takes a few seconds or much less. However, it could prove useful for nanotomography or laboratory CT scanners, for which a proper scan can take a few hours and the stage movement time is short compared with the radiographic exposure time.

Normalization and all shown preview reconstructions were processed and displayed directly during the scan on decent hardware (Intel Xeon W-2145 Workstation at 3.7 GHz with 256 GB RAM and an nVidia Geforce RTX 2080 Ti Graphics card with 11 GB VRAM). Only the complete volume reconstruction was carried out at the end of the scan within ∼12 min.

**Figure 11:**
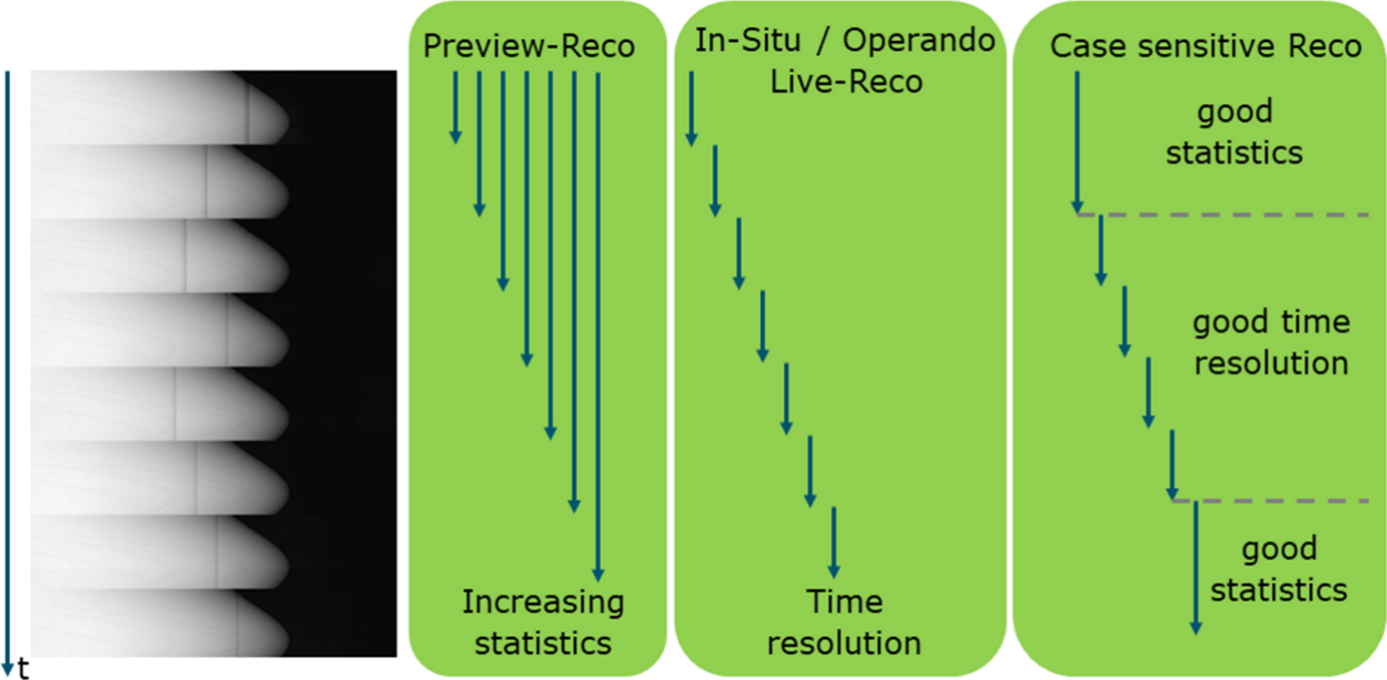
Additional potential options of sequence-based CT-scanning: Preview Reco as presented in this manuscript in which all sequences from the beginning contribute to a reconstruction. *In situ/operando* Live-Reco in which every sequence will be reconstructed as an individual (time step) volume reconstruction. Case sensitive Reco in which the number of sequences used for volume reconstruction depends on the needs of a case and the state of the sample.

The benefits of sequence-based scanning schemes extend beyond pure preview reconstructions and treatment of ring artifacts. It is possible to gain general flexibility when it comes to deciding about time resolution and image quality, which is usually done prior to XCT acquisition, *i.e.* when defining imaging parameters such as exposure times and number of projections. As depicted in Fig. 11 ▸, *in situ* and *operando* experiments could make use of sequence-based scans by exploiting the high time resolution and using only one or a few sequences for a volume reconstruction. As this ‘binning’ can be done flexibly after, or even during, an experiment, there is free choice of focusing on time resolution or on image quality. Possible experimental application could be in mechanical testing or battery cycling in which certain events cannot be predicted. Mechanical testing could be coupled with acoustic emission to identify the moment of crack initiation, which is important information about which sequences need to be included in a volume reconstruction. As the general trend in X-ray imaging is moving towards *in situ* and *operando* methods, laboratory-based XCT scanning systems will also have to adapt to this trend. The work presented here offers a cost-effective method for doing so.

## Supplementary Material

Video of Figure 8. DOI: 10.1107/S1600577526001177/tv5080sup1.mp4

Video of Figure 9. DOI: 10.1107/S1600577526001177/tv5080sup2.mp4

## Data Availability

All presented software is available via GitHub repositories (https://github.com/HMarkoetter/BAMline_CT_preview_ RECO and https://github.com/MichaS-git/BAMlineHelper).

## Footnotes

1 https://www.helmholtz-berlin.de/pubbin/igama_output?modus=einzel&sprache=de&gid=2469.

2 https://github.com/HMarkoetter/BAMline_CT_preview_RECO/tree/main/BAMline_step_scan_CT-reco.
